# The adult phenotype of Schaaf-Yang syndrome

**DOI:** 10.1186/s13023-020-01557-8

**Published:** 2020-10-19

**Authors:** Felix Marbach, Magdeldin Elgizouli, Megan Rech, Jasmin Beygo, Florian Erger, Clara Velmans, Constance T. R. M. Stumpel, Alexander P. A. Stegmann, Stefanie Beck-Wödl, Gabriele Gillessen-Kaesbach, Bernhard Horsthemke, Christian P. Schaaf, Alma Kuechler

**Affiliations:** 1 Institute of Human Genetics, Heidelberg University, Heidelberg, Germany; 2 Institute of Human Genetics, University Hospital Essen, University Duisburg-Essen, Essen, Germany; 3 Department of Molecular and Human Genetics, Baylor College of Medicine, Houston, TX USA; 4 Faculty of Medicine, University of Cologne, 50931 Cologne, Germany; 5 Institute of Human Genetics, University Hospital Cologne, Cologne, Germany; 6 Department of Clinical Genetics and GROW-School for Oncology and Developmental Biology, Maastricht University Medical Center, 6202AZ Maastricht, The Netherlands; 7 Institute of Medical Genetics and Applied Genomics, University of Tübingen, Tübingen, Germany; 8 Institute of Human Genetics, University of Lübeck, Lübeck, Germany; 9 Institute of Medical Genetics, University of Zurich, Zurich, Switzerland

**Keywords:** Schaaf-Yang syndrome, Prader–Willi syndrome, MAGEL2, Adult phenotype

## Abstract

**Background:**

*MAGEL2*-associated Schaaf-Yang syndrome (SHFYNG, OMIM #615547, ORPHA: 398069), which was identified in 2013, is a rare disorder caused by truncating variants of the paternal copy of *MAGEL2*, which is localized in the imprinted region on 15q11.2q13. The phenotype of SHFYNG in childhood partially overlaps with that of the well-established Prader–Willi syndrome (PWS, OMIM #176270). While larger numbers of younger individuals with SHFYNG have been recently published, the phenotype in adulthood is not well established. We recruited 7 adult individuals (aged 18 to 36) with molecularly confirmed SHFYNG and collected data regarding the clinical profile including eating habits, sleep, behavior, personal autonomy, psychiatric abnormalities and other medical conditions, as well as information about the respective phenotypes in childhood.

**Results:**

Within our small cohort, we identified a range of common features, such as disturbed sleep, hypoactivity, social withdrawal and anxiety, but also noted considerable differences at the level of personal autonomy and skills. Behavioral problems were frequent, and a majority of individuals displayed weight gain and food-seeking behavior, along with mild intellectual disability or borderline intellectual function. Classical symptoms of SHFYNG in childhood were reported for most individuals.

**Conclusion:**

Our findings indicate a high variability of the functional abilities and social participation of adults with SHFYNG. A high prevalence of obesity within our cohort was notable, and uncontrollable food intake was a major concern for some caregivers. The phenotypes of PWS and SHFYNG in adulthood might be more difficult to discern than the phenotypes in childhood. Molecular genetic testing for SHFYNG should therefore be considered in adults with the suspected diagnosis of PWS, if testing for PWS has been negative.

## Background

The *MAGEL2*-associated Schaaf-Yang syndrome (SHFYNG; OMIM #615547, ORPHA: 398069) is a rare hereditary neurodevelopmental disorder (prevalence < 1/1,000,000) with a profound impact on global development. The syndrome is usually evident at birth, with contractures of the interphalangeal joints, hypotonia and poor suck as characteristic early features. Global developmental delay usually becomes evident during infancy and intellectual disability of variable severity is present in all cases reported to date [1]. Hormonal anomalies such as hypogonadism and growth hormone deficiency are also part of the spectrum and some of the clinical features of SHFYNG mimic those observed in individuals with Prader–Willi Syndrome (PWS) [1–3]. PWS and SHFYNG are related disorders on the molecular level, as *MAGEL2* is among the genes located within the PWS critical region on 15q11.2-q13 [4–6]. The clinical picture of PWS is usually marked by neonatal hypotonia and poor suck in infancy, but children often develop hyperphagia and begin to gain weight from the third year of life onwards, with a high risk of morbid obesity and associated medical complications persisting throughout adulthood [7]. Unlike PWS, this hyperphagic phase is not a hallmark of SHFYNG in childhood, and has previously only been reported in 25% of individuals [1]. Developmental delay is usually more pronounced in children with SHFYNG compared to children with PWS, and criteria for autism spectrum disorder (ASD) are more frequently met (78% vs 26.7% [3, 8]). Both disorders are associated with clinical symptoms and variable degrees of disability in adulthood. While the phenotype of adults with PWS has been the subject of research over decades [9–12], clinical, social and behavioral traits of adults with SHFYNG have not been systematically studied. With the increasing availability of exome sequencing and its application in individuals with intellectual disability, the number of adults diagnosed with this disorder is likely to increase.

## Methods

### Patients and data collection

In this study, we investigated the phenotypes of 7 adult individuals with molecularly confirmed SHFYNG (individuals #1–7, who carry a truncating variant on the paternal *MAGEL2* allele) and one individual with the clinical phenotype of SHFYNG and a variant of unknown significance in *MAGEL2* (individual #8). We used a standardized questionnaire and, if available, clinical records from childhood onwards, with the aim to (1) characterize the adult phenotype of SHFYNG, (2) compare the adult phenotypes of SHFYNG and PWS, and (3) discern possible correlations between development in childhood and the phenotype in adulthood.

Individuals with SHFYNG and their families were recruited at the following institutions: University Hospital Cologne, Cologne (Germany), University Hospital Essen, Essen (Germany), Baylor College of Medicine, Houston (US), and Maastricht University Medical Center, Maastricht (the Netherlands). The study was also announced to members of the SHFYNG Facebook group (https://www.facebook.com/MAGEL2). Individuals with SHFYNG and their relatives and/or legal guardians from the United States (n = 4), Germany (n = 3), and the Netherlands (n = 1) expressed willingness to participate, and subsequently received the questionnaire. The legal guardians of all participants provided signed consent under the IRB-approved human research protocol H-34578, Baylor College of Medicine. Data regarding individuals #4 and #5 at a younger age had been previously published [1, 13].

### Molecular analysis

A description of the methods applied for molecular genetic testing in individuals #1–8 can be found in the Additional file 1.

## Results

**Fig. 1:**
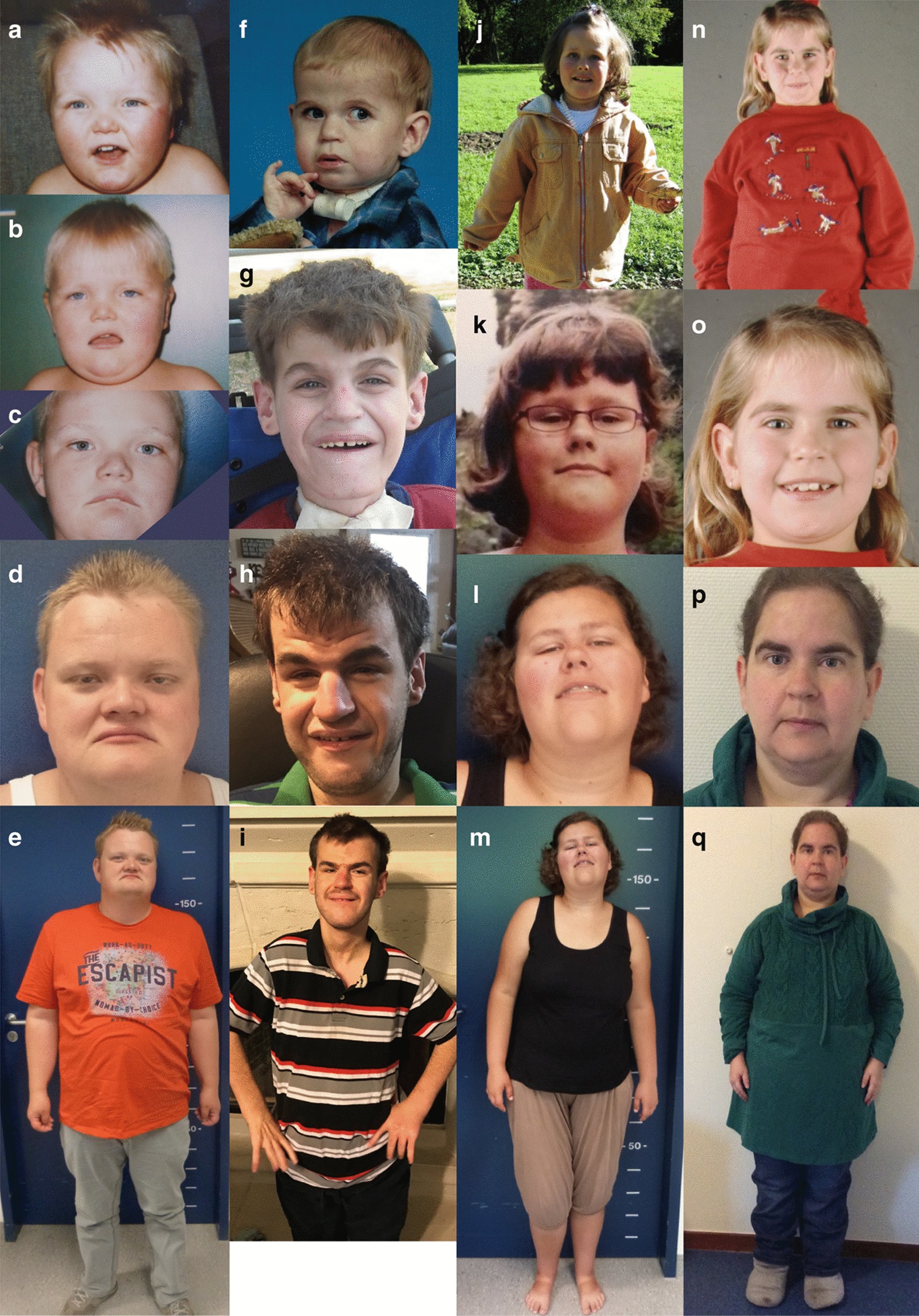
Clinical photographs of adult individuals with SHFYNG syndrome and evolution of the facial phenotype over time. Individual #2 at the age of 3 years (**a**), 5.5 years (**b**), 11.5 years (**c**) and 28 years (**d**, **e**). Individual #5 at the age of 2 years (**f**), 11 years (**g**), and 18 years (**h**, **i**). Individual #6 at the age of 5 years (**j**), 10 years (**k**), and 18 years (**l**, **m**). Individual #7 at age 9 (**n**, **o**), and 34 years (**p**, **q**)

**Fig. 2:**
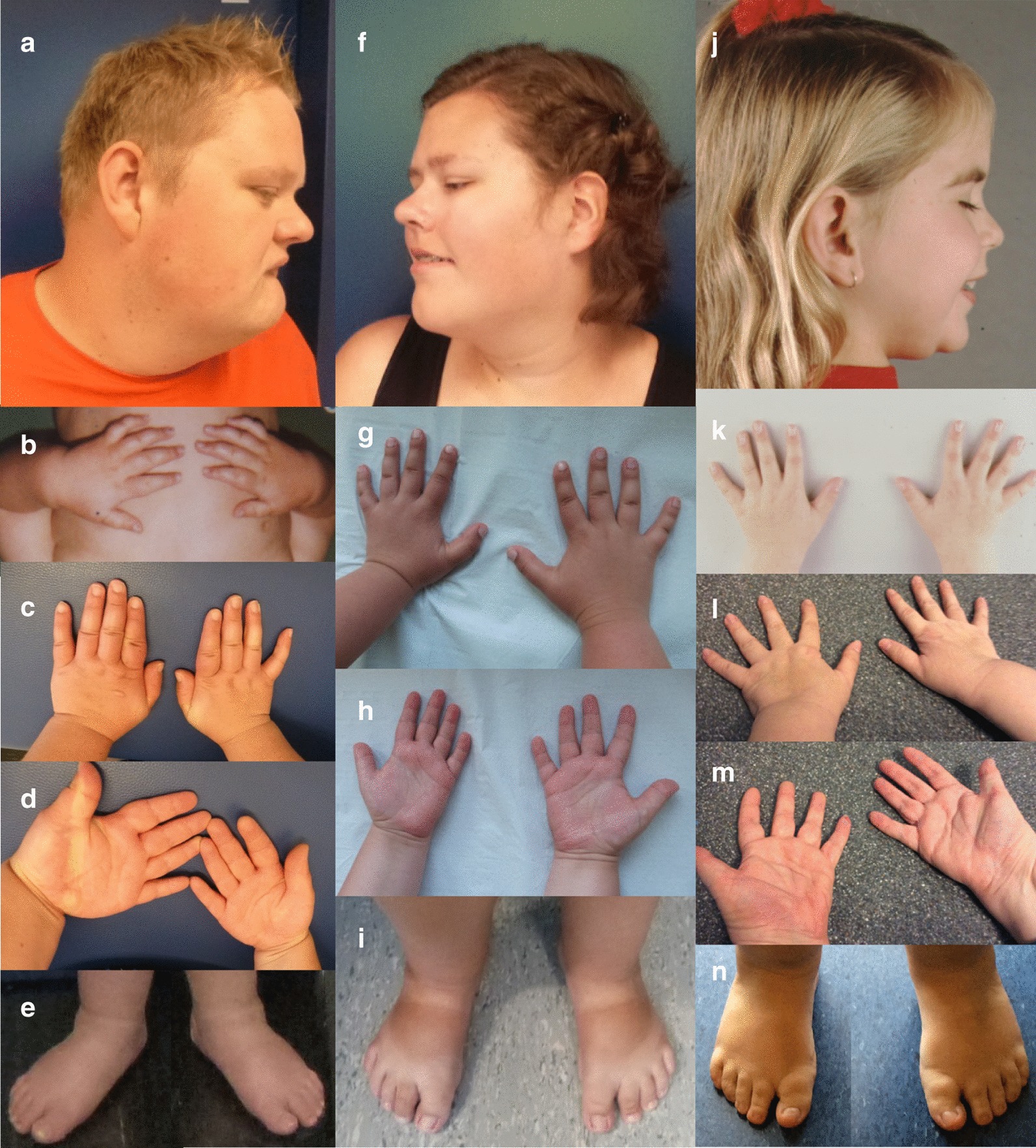
Clinical photographs of individuals with SHFYNG syndrome. Individual #2: facial profile at age 28 years (**a**), hands at age 3.5 years (**b**), hands and feet at age 28 years (**c**–**e**). Individual #6: facial profile, hands and feet at age 18 years (**f**–**i**), Individual #7: facial profile and hands at age 9 (**j**, **k**), hands and feet at age 34 years (**l**–**n**)

Adult individuals and their respective family members and/or legal guardians received a questionnaire (see Additional file 1) which was divided into sections including eating habits, behavior, autonomy, as well as psychiatric and other medical conditions. Clinical records from childhood were also collected and compiled (Table 1). The questionnaire can be found in the Additional file 1. compilation of clinical photographs of individuals #2, #5, #6, and #7 can be found in Figs. 1 and 2. We excluded individual #8 from the compilation, as the diagnosis of SHFYNG, while likely, could not be molecularly confirmed.

**Table 1:** Overview of the reported phenotypic features of individuals #1–#8 in childhood, as well as the respective phenotypes in adulthood including food intake, behavior, autonomy, and medical conditions

Individual #	1	2	3	4	5	6	7	8
Age (years)	25	30	26	20	18	18	36	55
Gender	F	M	F	M	M	F	F	M
*MAGEL2* variant (NM_019066.4)	c.2874G > A p.Trp958*	c.1800delT p.Pro601Glnfs*101	c.1819C > T p.Gln607*	c.2958delG p.Ser987Alafs*5	c.1996dupC p.Gln666Profs*47	c.1790delC p.Pro597Hisfs*105	c.2821dupC p.Arg941Profs*10	c.2170_2232dup p.Ser724_Ala744dup
Inheritance	De novo	De novo	Paternal	De novo	Paternal	Unknown	Unknown	Unknown
Height (cm)	144.8	170	n.s	175.9	148	174	147	160
BMI (kg/m^2^)	31.4	41.2	n.s	30.6	22	31.7	32	51.6
Head circumference (cm)	n.s	56	n.s	n.s	n.s	54	55	57
IQ	Mild intellectual disability	Mild intellectual disability	n.s./n.a	Learning disability (WAIS-IV full scale: 84)	n.s	Learning disability (74–85)	n.s	Mild intellectual disability (WAIS-IV: 67)
*Phenotype in childhood*								
Birth weight (kg)	2.35	3.33	2.61	n.s	2.89	3.42	3.28	n.s
Birth length (cm)	n.s	50	n.s	n.s	48.3	54	46	n.s
Head circumference (cm)	n.s	36.5	32.7	n.s	n.s	36	n.s	n.s
Gestational week	35	40	35	n.s	n.s	40	38	n.s
Decreased fetal movement	n.s	Yes	n.s	n.s	n.s	Yes	n.s	n.s
Congenital contractures of interphalangeal joints	Yes	Yes	Yes	Yes	Yes	No	Yes	n.s
Neonatal hypotonia, poor suck	Yes	Yes	Yes	Yes	Yes	Yes	No	n.s
Feeding problems in infancy	Yes	Yes	Yes	Yes	Yes	Yes	No	Yes
Nasogastric tube feeding	n.s	Yes	Yes	n.s	Yes	Yes, for 1 mo	No	n.s
Motor development	Delayed	Delayed	Severely delayed, never walked	Delayed	Delayed	Delayed	Delayed	Delayed
Language development	Delayed	Delayed	None	Delayed	Delayed	Normal	n.s	n.s
Speech, articulation defects	n.s	Fluent, mild (sometimes slurred speech)	n.a	n.s	First words at 11 years of age	Fluent	Strong verbal qualities	Fluent, mild
Excessive weight gain before the age of 6 years	n.s	Yes	No	No	No	Yes	n.s	Possibly Yes
*Phenotype in adulthood*								
Food intake, metabolism and sleep (− = never; + = 1–3 times/month; ++ = 1–3 times/week; +++ = 4–7 times/week)								
Overeating	+++	+++	n.a. (PEG tube)	+++	−	+	+	+++
Food-seeking behavior	++	+++	n.a	+++	−	+	+	++
Sleep apnea	+	+++	−	+++	+++	−	n.s	+++
Need for assisted ventilation (CPAP, BIPAP)	−	+++	−	+++	+++	−	−	+++
Abnormal sleep cycle^a^	++	++	+++	+	+++	+	++	+++
Excessive sleeping/daytime fatigue	+++	++	+++	+++	+++	−/+	−	++
Constipation	+++	++	−	++	+++	+	−	+++
Behavior and psyche (− = not present and/or not an issue; + = infrequent and/or not a big issue; ++ = frequent and/or sometimes a big issue;+++ = almost always present and/or a major concern)								
Hyperactivity	++	−	−	+	−	−	−	++
Underactivity	++	+++	−	++	n.s	+++	++	+++
Stubbornness	+++	++	−	++	++	+	++	+++
Temper tantrums	+	+	−	++	−	−	−	++
Aggression	+	−	−	−	−	−	−	++
Lying, deceitfulness	+++	−	n.a	++	−	−	+	+
Stealing	+++	−	n.a	++	−	−	+	++
Manic, excited mood	+++	++	−	−	−	−	−	++
Depressive mood	+	++	−	+	−	−	−	+++
Anxiety	++	+	−	++	+	+++	+	+++
Social withdrawal	+++	+	n.a	+	−	+	+	+++
Restricted interests	+++	n.s	n.a	++	++	++	+	++
Self-stimulatory behavior	+	+++	−	−	++	−	−	+
Skin picking	+++	−	−	+	−	−	+	+++
*Autonomy and activities*								
Communication	Verbal	Verbal	None	Verbal	Mostly nonverbal, ≤ 50 Signs/gestures	Verbal	Verbal	Verbal
Reading skills	Basic	Not present	Not present	Basic	Basic	Good	Good	Good
Awareness of danger	Severely reduced	Reduced	Severely reduced	Reduced	Severely reduced	Severely reduced	Reduced	Normal
Can take care for basic needs^b^	Yes, but help is required in certain areas	Sometimes, but help is required regularly	No	Yes, but help is required in certain areas	No	With help	Yes, but help is sometimes required	Yes
Performs basic (housekeeping) chores^c^	No/mostly not	Sometimes	No	Regularly	No	No/mostly not	Sometimes	Sometimes
Performs more complex (everyday) activities^d^	No/mostly not	No/mostly not	No	Sometimes	No	No/mostly not	Regularly	Sometimes
Sports/physical activities	No/mostly not	Regularly	No	Sometimes	Sometimes	Occasionally	No	No
Works (e.g. in a sheltered environment)	No/mostly not	Regularly	No	Regularly	No	Regularly	Regularly	No
*Medical conditions, medication*								
Hypothyroidism	No	Yes	No	Yes	No	No	No	Yes
Hypogonadism^e^	n.s	Yes	Yes	No	No	No	No	Yes
Type 2 diabetes	No	No	No	No	No	No	No	No
Hypertension	No	No	No	No	No	No	No	Yes
Scoliosis	Yes	Yes	Yes	No	Yes	No	Yes	No
Autism spectrum disorder	Yes	No	n.a	Yes	Yes	Yes (Rocking body movements)	No	No
Attention deficit disorder	Yes	No (?)	n.a	n.s	n.s	n.s	No	n.s
Obsessive compulsive disorder	Yes	No (?)	n.a	Yes	n.s	n.s	No	No
Growth hormone therapy	Yes	No	No	No	Yes	No	No	No
Use of melatonin	No	No	No	Yes	No	No	No	No

*n.s.* not specified, *n.a.* not applicable

^a^A sleeping routine that is different from the normal sleep cycle (e.g. sleeping from 2 pm to 1 am). A “reverse” sleep cycle would be the most extreme form

^b^E.g. getting dressed, using the toilet, basic body hygiene

^c^E.g. cleaning his or her room, preparing food

^d^E.g. buying groceries, or using public transport

^e^Defined as repeatedly low levels of sex hormones

Phenotype in childhood

Most individuals of our cohort had a typical presentation of SHFYNG at birth, with neonatal hypotonia, feeding difficulties and contractures of the interphalangeal joints in 6 out of 7 individuals. Motor and language development was delayed in all cases, and 2 out of 7 individuals displayed excessive weight gain before the age of 6 years. Notable were behavioral difficulties already in childhood with frequent tantrums, anger outbreaks, social withdrawal, and in at least one case aggressive behavior during puberty. All patients demonstrated mild intellectual disability to low normal intelligence.

Our data regarding the adult phenotype of patient #1–7 can be subdivided into 4 main areas:

1. Food intake, body weight and sleep

Among the most challenging symptoms for both individuals and their family members was excessive weight gain, leading to obesity (BMI > 30 kg/m^2^) in 5 out of 7 individuals. Overeating and food-seeking behavior was present in all individuals with obesity. Constipation of varying severity, a frequent feature in children with SHFYNG [1], was present in 5 out of 7 adult individuals except for individual #7 and individual #3 (who has an intragastric PEG/ MIC-KEY tube).

A disturbed sleep cycle with daytime fatigue was reported throughout our cohort, and reports ranged from trouble sleeping through, which is present to variable extent in all individuals, to a completely disorganized sleep pattern in the case of individual #3. Sleep apnea was reported in 4 individuals, necessitating assisted ventilation during night-time for 3 out of 4 individuals. While increased BMI is linked to a higher prevalence of obstructive sleep apnea in the general population, the presence of sleep apnea does not exclusively correlate with obesity in our cohort. This finding is in accordance with a high rate of obstructive as well as central sleep apnea reported in younger SHFYNG individuals, the majority of whom did not display excessive weight gain at the time of data collection [1].

2. Behavioral phenotype

Formal intelligence testing, if performed, produced results compatible with mild intellectual disability (ID) or low-normal intelligence, although no formal testing of the more severely affected individuals had been performed. The majority of individuals in our cohort showed features associated with autism spectrum disorder (ASD) with restricted and sometimes obsessive interests reported in 5 individuals. These interests included relatively simple obsessions like a fascination with insects, as well as more complex activities such as computer games. Social withdrawal was reported in 5 out of 7 individuals, and 3 individuals displayed self-stimulatory behavior. Another prominent behavioral feature was lack of activity and initiative (5 individuals). While stubbornness (6 individuals) and mostly occasional temper tantrums (3 individuals) were reported, most individuals did not show aggressive behavior. Infrequent bouts of aggression in response to an external restriction of obsessive interests, or unwanted changes of environment, were only reported in individual #1. It is also notable that 6 out of 7 individuals suffered from anxiety of varying severity. In the case of individual #4, episodes of high anxiety preceded the onset of common respiratory infections.

3. Autonomy and activities

The capabilities of individuals in our cohort differed considerably. While 5 individuals communicated verbally, individual #5 relied on signs and gestures, and no systematic mode of communication had been established in the case of individual #3. Additionally, 5 individuals had at least basic reading skills. All individuals required some external help with basic self-care such as dressing and body hygiene, and 2 individuals (#3 and 5) were fully reliant on external help in their daily life. Concerning everyday activities, 3 individuals (# 2, 4 and 7) performed housekeeping chores, while individuals #4 and #7 engaged in several more complex tasks. A total of 4 individuals participated in sports or physical activities to some extent. Additionally, 4 individuals worked in a protected environment on a regular basis. All individuals lived in specialized institutions or at home with their families. A reduced or severely reduced awareness of danger was reported in all individuals of our cohort.

4. Medical conditions

Neither type 2 diabetes nor hypertension was reported in any of the 5 individuals with BMI > 30 kg/m^2^ (individuals # 1, 2, 4, 6 and 7, aged 18–36 years). Individual #2, for whom overeating and obesity had been a constant concern since childhood, had normal fasting glucose and insulin levels, as well as a normal oral glucose tolerance test at the age of 19 years (BMI 34.1 kg/m^2^). Repeated measurements of fasting glucose in individual #6 also produced normal results. Hypothyroidism was noted in individuals #2 (30 years) and #4 (20 years) of our cohort. This feature, which is present in roughly 25% of individuals with PWS [5], had not been previously found in younger individuals with SHFYNG [3] (aged 0.5–18 years). Hypogonadism was observed in 2 individuals. Scoliosis was present in 5 individuals, and individual #3 showed evidence for bone fragility (repeated fractures of the femur). Individuals #1 and #5 received growth hormone therapy, and individual #4 melatonin therapy for the treatment of a sleeping disorder. ASD was diagnosed in 4, and obsessive–compulsive disorder (OCD) was diagnosed in 2 individuals. Individual #6 was diagnosed with paranoid schizophrenia, which was treated with antipsychotic medication (Aripiprazole and Amisulpride).

Individual #8

Individual #8 first presented at the age of 55 years. Clinical records indicated feeding difficulties in infancy, developmental delay and learning difficulties, as well as the onset of hyperphagia in childhood. The patient presented with mild intellectual disability, and personnel of his specialized institution reported behavioral problems including bouts of aggression, chronic skin picking and uncontrolled eating. He had been hospitalized once due to a major depressive episode. Clinical examination revealed low height (160 cm), obesity (BMI 51.6), and hypoplastic genitalia. He had tapering fingers with scarred finger tips. The clinically suspected diagnosis of PWS could not be molecularly confirmed by chromosome array-comparative-genomic-hybridization or methylation-sensitive MLPA. Targeted sequence analysis of *MAGEL2* then revealed an in-frame duplication of 63 base pairs (c.2170_2232dup; p.(Ser724_Ala744dup). This variant was located on the paternal allele (confirmed by methylation-sensitive testing), but was classified as a variant of unknown significance, as only frameshift- or stop-variants of *MAGEL2* have so far been reported as pathogenic, and it is present 2 times in gnomAD v3 [14]. Additional genetic testing such as exome sequencing had not been performed, and family members were not available for segregation analysis. Regarding the presence of the variant c.2170_2232dup in gnomAD, it should be noted that it is unknown if the respective individuals carry the variant on the paternal or the maternal allele. Since only pathogenic variants of *MAGEL2* which are located on the paternal allele will cause the SHFYNG phenotype, pathogenic frameshift or stop variants of this gene can be found at extremely low frequencies in the gnomAD v2.1.1 and v3 datasets (see Web resources), which represent a (mostly) healthy control population. As individual #8 presented with hallmark symptoms of SHFYNG, we nevertheless suspect this diagnosis to be present. An overview of the phenotype of individual #8 can be found in Table 1.

## Discussion

Schaaf-Yang syndrome was initially described in patient cohorts consisting predominantly of children, while adults with SHFYNG have been underrepresented. Adult individuals with *MAGEL2* mutations have been reported in the literature, such as the cases presented by Jobling et al. (individual I-1) [15], or Fountain et al. (individual 18) [13], but a systematic investigation of adults with this disorder has not been done previously. The phenotypes observed in these individuals are highly variable, especially with regard to the degree of personal independence and social participation, ranging from semi-autonomous living (individual #4) to complete dependence on external help (individual #3). However, most individuals acquired verbal communication skills and participated in social activities like sports or games, and 4 out of 7 individuals worked on a regular basis, albeit in protected work environments. Major concerns of the relatively high-performing individuals and their families were overeating and subsequent weight problems, although complications like hypertension and type 2 diabetes were yet to manifest. Reduced activity and fatigue negatively impacted the daily life of most individuals, and heightened anxiety was reported almost unanimously among the cohort. Additionally, the prevalence of psychiatric disorders (ASD, OCD, paranoid schizophrenia) was high (4 out of 7 individuals). There was a general trend towards an exacerbation of social withdrawal and behavioral abnormalities with progressing age. The pubertal period proved particularly testing for families and caregivers.

The cognitive ability of individuals of our small cohort was mostly within the range of mild intellectual disability or borderline intellectual function, which is inconsistent with previous findings from a larger cohort of younger individuals, where the average IQ was within the range of moderate intellectual disability [1]. Possible explanations for this might be the small sample size, or the presence of a selection bias, for example due to a higher overall childhood mortality in more severely affected individuals.

A notable similarity between adults with SHFYNG and PWS is a high prevalence of obesity and food-seeking behavior, although this symptom is not present in all individuals of our cohort. Overeating could be observed in individuals with a higher degree of independence and verbal communication skills (individuals # 1, 2, 4, 6 and 7), and it is absent in the individuals who are in need of external help for basic self-care and where verbal communication is not possible (individuals # 3 and 5).

Children with SHFYNG display a characteristic combination of symptoms, with marked differences between SHFYNG and PWS. These include the prevalence of contractures at birth, a more pronounced developmental delay, a lower rate and later onset of hyperphagia in childhood, and a higher prevalence of autistic features [1, 3, 16]. The onset of obesity in individuals with SHFYNG, while highly variable, seems to occur generally later in life than the onset of obesity in individuals with PWS (6–12 years [6]). Given the generally slower global development and lower cognitive ability of individuals with SHFYNG compared to PWS [1, 5, 17], one possible explanation for this observation might be an inability to independently acquire food rather than a lower appetite. Food intake may therefore be better controllable in some individuals during childhood, and may exacerbate later in life once the individual’s skills and cognitive ability allow it. In this case, the onset of obesity would be the consequence of a milder phenotype with greater cognitive abilities. Alternatively, a greater desire for food might simply accompany (and be indicative of) a milder, more PWS-like, course of SHFYNG. Both explanations would be compatible with the presence of obesity in adults with higher cognitive function, and its absence in more severely affected individuals within our small cohort.

Another emerging connection between SHFYNG and PWS in adulthood might be an increased risk of psychiatric disorders. Based on a study of 70 adults with PWS living in residential care by Manzardo et al. [18] (mean age 37 ± 10 years), anxiety disorders were reported in 38%, and psychotic features including schizophrenia had been diagnosed in 23%, while mood disorders were also prevalent (major depressive disorder in 24%, nonpsychotic bipolar disorder in 21%). The small cohort size of our study does not allow a definitive risk assessment, but the presence of anxiety in all but one individual, and paranoid schizophrenia in 1 individual, may indicate a similar pattern of psychiatric disorders in adults with SHFYNG.

## Conclusions

**Fig. 3:**
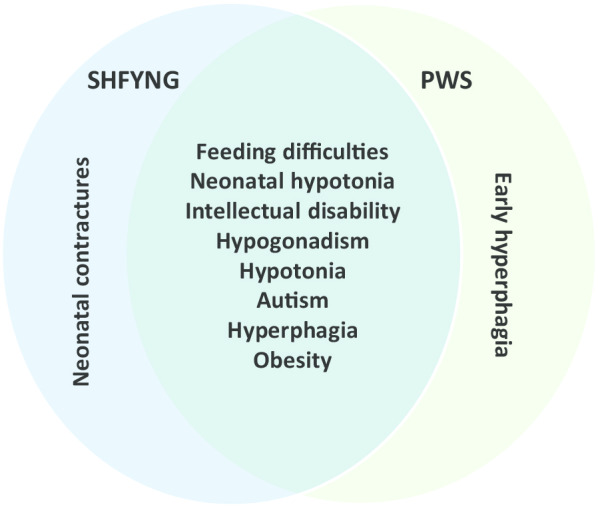
Venn diagram of features of adults with SHFYNG in our cohort and major features of PWS. This figure is based on the Venn diagram originally published by Fountain et al. ([16], Figure 1)

Our assessment of adults with SHFYNG demonstrated a high variability of behavioral characteristics and functional abilities. The level of autonomy ranged from complete dependence on external help to semi-autonomous living, and cognitive ability was mostly classified as mild intellectual disability or borderline intellectual function. All individuals had behavioral problems and a lack of activity and motivation were frequent, as well as abnormalities of the sleep cycle and fatigue. Obesity was present in a majority of cases, and almost all participants had the typical phenotype of SHFYNG in childhood. Hallmark symptoms of PWS in adulthood were present in most individuals, which makes the distinction between adults with SHFYNG and those with the more frequent PWS more difficult. In those cases, information regarding the phenotype in childhood (such as contractures at birth, more severely delayed development, later onset of hyperphagia), if available, might be the most reliable anamnestic tool. To illustrate the large phenotypic overlap of both disorders, the most prominent features of SHFYNG and PWS in adults are shown in Fig. 3. The differential diagnosis of SHFYNG should be considered in every adult with the suspected diagnosis of PWS, if molecular genetic testing for PWS is negative. Similar to adults with PWS, adults with SHFYNG might have a significantly higher risk of psychiatric disorders.

### Supplementary information

**Additional file 1.** Supplementary methods and questionnaires.

## Data Availability

The dataset supporting the conclusions of this article is included within the article (and its additional file).

## Web resources

Genome Aggregation Database (gnomAD)—annotated variants of the *MAGEL2* gene:

- https://gnomad.broadinstitute.org/gene/ENSG00000214107?dataset=gnomad_r2_1
- https://gnomad.broadinstitute.org/gene/ENSG00000254585?dataset=gnomad_r3

## Abbreviations

ASD: Autism spectrum disorder
BMI: Body Mass Index
*MAGEL2*: MAGE family member L2
MLPA: Multiplex ligation-dependent probe amplification
OCD: Obsessive-compulsive disorder
PWS: Prader–Willi syndrome
SHFYNG: Schaaf-Yang syndrome
