# Supplementary material for: The adult phenotype of Schaaf-Yang syndrome
Source: Orphanet J Rare Dis. 2020 Oct 19;15:294. doi: 10.1186/s13023-020-01557-8 (PMC7574436; doi:10.1186/s13023-020-01557-8)
Supplement: Supplementary file 1 — Additional file 1. Supplementary methods and questionnaires. [file 13023_2020_1557_MOESM1_ESM.docx]

**Supplementary material**

1. Supplementary Methods Page **2**
2. Questionnaire for families/patients/legal guardians Page **4**
3. Childhood Questionnaire Page **10**

**Supplementary Methods**

Molecular genetic analysis of individual #1 - #8

**Individual #1** received exome sequencing (GeneDx, Gaithersburg, MD, USA). Genomic DNA was enriched for coding regions and splice-site junctions of most genes using a proprietary capture system developed by GeneDx for next-generation sequencing with CNV-calling. The enriched targets were simultaneously sequenced with paired-end reads on an Illumina platform. Bi-directional sequence reads were assembled and aligned to the human genome build GRCh37/UCSC hg 19 and analyzed by a custom-developed analysis tool (XomeAnalyzer).

**For individual #2,** exome sequencing was performed on genomic DNA. Coding genomic regions were enriched with a SureSelect Human All Exon Kit V6 (Agilent technologies, Santa Clara, California) for subsequent sequencing as 2x125 bp paired-end reads on an HiSeq2500 system (Illumina, San Diego, California). Whole exome sequencing (WES) for the index case resulted in an average 147-fold coverage with 95.8 % of the target sequences being covered at least 20-fold. Parental analysis was performed by Sanger sequencing.

**Individual #3** received whole exome sequencing under the umbrella of the Deciphering Developmental Disorders (DDD) study. The presence of a pathogenic *MAGEL2* variant was then confirmed by molecular genetic testing in a diagnostic setting (Cinical Genetics Service, Nottingham University Hospitals, Nottingham, UK). We received no further information regarding the test methods.

**Individuals #4** and **#5** were diagnosed by clinical exome sequencing at the Baylor Genetics Laboratories (Houston, USA). Briefly, genomic DNA samples were fragmented, ligated to Illumina multiplexing paired-end adaptors, amplified with indexes added, and hybridized to a solution-based exome capture reagent (Roche NimbleGen). Paired-end sequencing (2 × 100 bp) was performed on the Illumina HiSeq 2500 platform to provide a mean sequence coverage of about 120×, with about 97% of the target bases having at least 20× coverage. Exome sequencing data were processed and variants were annotated as previously described (Yang et al., 2013) [PMID 24088041]. Briefly, the output data from the Illumina HiSeq 2500 were converted from a bcl file to a FastQ file by Illumina Consensus Assessment of Sequence and Variation software version 1.8.3 and mapped to the human reference genome using the BWA program23. Variants were called by Atlas-SNP and Atlas-indel24. An in-house software program, CASSANDRA, was used for variant filtering and annotation.

For **individuals #6** and **#8**, targeted sequence analysis of *MAGEL2* was performed by longrange PCR followed by Sanger sequencing using standard protocols. The fragment was sequenced on an ABI 3130XL sequencer (Applied Biosystems, Darmstadt, Germany). Data analysis was conducted with the Sequencing Analysis v7 (Applied Biosystems) and the Geneious software (Biomatters, Auckland, New Zealand). In the case of individual #8 had, Sanger sequencing of *MAGEL2* using standard methods had been performed beforehand.

Whole exome sequencing and variant calling of **Individual #7** was performed as previously described (De Ligt et al., 2012) [PMID 23033978]. Briefly, the exome was captured using the Agilent SureSelectXT Human All Exon v5 library prep kit (Agilent Technologies, Santa Clara, CA, USA) and exome libraries were sequenced on an Illumina HiSeq 4000 instrument (Illumina, San Diego, CA, USA). The reads were aligned to the hg19 reference genome using Burrows‐Wheeler Alignment version 0.5.9‐r16.14. The Genome Analysis Toolkit (GATK) unified genotyper (version 3.2‐2) was used for variant calling. Variants were annotated using a custom diagnostic annotation pipeline and classified adhering to ACMG standards and guidelines.

**Methylation-sensitive testing** was performed based on a previous description (Schaaf et al. 2013) in individuals #2, #6, and #8 to proof localization of the detected *MAGEL2* variants on the paternal allele. Briefly, DNA from blood was digested using the methylation-sensitive restriction enzyme SmaI (New England Biosystems, Frankfurt, Germany) cleaving the region of *MAGEL2* on the unmethylated paternal allele. Afterwards, longrange PCR followed by Sanger sequencing was conducted as described above.

**Schaaf-Yang Syndrome in adulthood**

**Questionnaire for families/patients/legal guardians**

Dear participants,

The following questionnaire aims to provide an oversight over the different aspects of Schaaf-Yang-Syndrome/SYS in adults. It covers several areas of interest including metabolism, abilities and activities, and medical conditions. We intend to use the information you provide to further the understanding of the clinical spectrum of adults with SYS, and we will therefore aim to publish a compilation of the information collected from several families in an appropriate scientific journal. This process will include anonymization of the data (i.e. removal of names, dates). The questionnaire is composed of 6 sections featuring “yes” or “no” questions and checkboxes, as well as text boxes for comments or further explanations. It will take approximately 15-30 minutes to complete. Please **only check one box for every question**.

After completion of the form, please send it via email to **Megan.Rech@bcm.edu**. You will then receive a consent form to the ongoing study "Understanding the Molecular Causes of Neuropsychiatric Disease" (H-34578) of the Baylor College of Medicine. We will also ask for a copy of the genetic test result of your affected relative. The information provided in this questionnaire will only be used *after* we have received your formal consent. Your contribution is highly appreciated and we would like to thank you very much for taking the time to participate.

Sincerely,

Dr. Felix Marbach Prof. Dr. Christian P. Schaaf

**Section 1 - General information**

Age:

Gender:

Height: Date of measurement:

Weight: Date of measurement:

IQ (if standardized testing has been performed):

# Section 2 - Food intake, metabolism and sleep

| **Frequency** | Never/not  applicable | Infrequent (1-3  times/month) | Frequent (1-3  times/week) | Common (4-7  times/week) |
| --- | --- | --- | --- | --- |
| Overeating | □ | □ | □ | □ |
| Food-seeking behavior | □ | □ | □ | □ |
| Not eating enough/  disinterested in food | □ | □ | □ | □ |
| Normal food intake | □ | □ | □ | □ |
| Constipation | □ | □ | □ | □ |
| Diarrhea | □ | □ | □ | □ |
| Sleep apnea | □ | □ | □ | □ |
| Use of CPAP/BIPAP  during sleep | □ | □ | □ | □ |
| Trouble sleeping  through* | □ | □ | □ | □ |
| Abnormal sleep  cycle** | □ | □ | □ | □ |
| Daytime fatigue | □ | □ | □ | □ |
| Excessive sleeping  (12+ hrs.) | □ | □ | □ | □ |
| Regular sleep*** | □ | □ | □ | □ |

* Waking up equal or more than 4 times during the sleep cycle.

** A sleeping routine that is different from the normal sleep cycle (e.g. sleeping from 2pm to 1am). A “reverse” sleep cycle would be the most extreme form.

*** 6-10 hrs. of sleep largely during night-time, waking up less than 4 times during the sleep cycle. Additional comments:

# Section 3 - Autonomy and activities

Awareness of danger/personal safety □Normal □Reduced □Severely reduced Needs help using the toilet □Regularly □Sometimes □ Not or mostly not

Needs help dressing □Regularly □Sometimes □Not or mostly not Needs help with basic body hygiene □Regularly □Sometimes □ Not or mostly not Performs basic housekeeping chores* □Regularly □Sometimes □ Not or mostly not Performs more complex (everyday) activities** □Regularly □Sometimes □ Not or mostly not Partakes in games/ playful activities □Regularly □Sometimes □ Not or mostly not Partakes in sports/ physical activities □Regularly □Sometimes □ Not or mostly not Works (e.g. in a sheltered workshop) □Regularly □Sometimes □ Not or mostly not Reading skills □Good □Basic □Not present Communication □Verbal □>10 Signs/gestures □≤10 Signs/gestures □None Can be alone for… □Not at all □≤1hrs. □1-8 hrs. □>8 <24 hrs. □>24 hrs.

Residency: □At home with family □Specialized institution □Semi-autonomous/assisted living

- E.g. cleaning his or her room, preparing food

** E.g. using public transportation, buying groceries Additional comments/explanations:

**Section 4 - Behavior and psyche**

**1** = Not present and/or not an issue; **2** = Infrequent and/or not a big issue; **3** = Frequent and/or sometimes a big issue; **4** = Almost always present and/or a major concern.

| **Scale (1 – 4)** | **1** | **2** | **3** | **4** |
| --- | --- | --- | --- | --- |
| Hyperactivity/overactivity | □ | □ | □ | □ |
| Underactivity | □ | □ | □ | □ |
| Stubbornness | □ | □ | □ | □ |
| Temper tantrums | □ | □ | □ | □ |
| Aggression towards  others | □ | □ | □ | □ |
| Intentionally destructive  behavior** | □ | □ | □ | □ |
| Intentionally disruptive  behavior | □ | □ | □ | □ |
| Inappropriate sexual  activity | □ | □ | □ | □ |
| Lying, deceitfulness | □ | □ | □ | □ |
| Stealing (food, objects  etc.) | □ | □ | □ | □ |
| Manic, excited mood | □ | □ | □ | □ |
| Gloomy, depressive mood | □ | □ | □ | □ |
| Recurring pronounced  changes of mood | □ | □ | □ | □ |
| Anxiety | □ | □ | □ | □ |
| Withdraws from other  people | □ | □ | □ | □ |
| Seeks human contact  inappropriately | □ | □ | □ | □ |
| Problems with small  changes in routine | □ | □ | □ | □ |
| Self-stimulatory behavior  *** | □ | □ | □ | □ |
| Restricted interests,  obsessions | □ | □ | □ | □ |
| Skin picking | □ | □ | □ | □ |
| Other self-mutilating  behavior | □ | □ | □ | □ |

** E.g. intentionally breaking objects.

***Repetitive actions like hand flapping, rocking, or noises, sometimes in response to overstimulation.

Additional comments/specifications:

# Section 5 - Medical conditions, medication

Hypothyroidism (underactive thyroid) □Yes □No Hypogonadism (repeatedly low levels of sex hormones) □Yes □No Type 2 diabetes □Yes □No

Hypertension (abnormally high blood pressure) □Yes □No

Hypotonia (abnormally low muscle strength) □Yes □No

Evidence of bone fragility* □Yes □No

Scoliosis □Yes □No

Growth hormone therapy** □Yes □No Use of Melatonin for the treatment of sleep disorders □Yes □No Other medication (*please specify below*) □Yes □No

- Multiple fractures (>2), or fractures after inadequate trauma - *please specify below*.

** *Please specify the age at which the therapy was initiated and the duration of the therapy.*

Comments or *specifications*:

# Section 5 - Medical conditions, medication

Has an additional medical condition been diagnosed? □Yes □No If the answer is yes, you may choose to specify:

Has a psychiatric disorder been diagnosed? □Yes □No If the answer is yes, you may choose to specify:

**Childhood Questionnaire**

Please describe in a few words the symptoms or anomalies which were evident during childhood. This includes anomalies of the body (such as contractures, short stature or obesity), as well as information regarding development of language and motor skills, and behavioral traits (such as excessive eating or autism).

**Weight, body length and head circumference at birth:**

|  |
| --- |

**Anomalies at birth:**

E.g. Premature birth, contractures, muscular hypotonia, poor suck, small for gestational age etc.

|  |
| --- |

**Development during the neonatal period and infancy (first year of life):**

E.g. development of muscle strength, onset of independent feeding, general motor development etc.

|  |
| --- |

**Development from one to six years:**

E.g. age at which the child was crawling, began walking independently, spoke the first words etc. Onset of (increased) interest in food? Evidence for autistic traits/features?

|  |
| --- |

**Age six to 12 years:**

E.g. onset of excessive eating, diagnosis of autism, language development, development of body weight etc.

|  |
| --- |

**Age 13 years to adulthood:**

|  |
| --- |
